# Propofol Infusion Syndrome in Refractory Status Epilepticus: A Case Report and Topical Review

**DOI:** 10.1155/2016/3265929

**Published:** 2016-07-14

**Authors:** Akil Walli, Troels Dirch Poulsen, Mette Dam, Jens Børglum

**Affiliations:** Department of Anaesthesia and Intensive Care Medicine, Zealand University Hospital, University of Copenhagen, Sygehusvej 10, 4000 Roskilde, Denmark

## Abstract

Propofol infusion syndrome (PRIS) is a fatal complication when doses of propofol administration exceed 4 mg/kg/h for more than 48 hours. Propofol overdosage is not uncommon in patients with refractory status epilepticus (RSE). We describe a case of refractory status epilepticus complicated by propofol infusion syndrome and collect from 5 databases all reports of refractory status epilepticus cases that were treated by propofol and developed the syndrome and outline whether refractory status epilepticus treatment with propofol is standardized according to international recommendations, compare it with alternative medications, and discuss how this syndrome can be treated and prevented. A total of 21 patients who developed this syndrome reported arrhythmia in all cases (100%), rhabdomyolysis in 9 cases (42%), lactic acidosis in 13 cases (62%), renal failure in 8 cases (38%), lipemia in 7 cases (33%), and elevated hepatic enzymes in 6 cases (28%). 13 patients died (66%). Propofol is still given in a dosage higher than what is internationally recommended, and new treatment modalities such as renal replacement therapy, blood exchange, and extracorporeal membrane oxygenation seem to be promising. In conclusion, propofol should be carefully titrated, the maximal infusion rate needs to be reassessed, and combination of different sedative agents may be considered.

## 1. Introduction

Propofol infusion syndrome is a rare, usually fatal complication of prolonged propofol infusion for more than 48 hours with a dosage of more than 4 mg/kg/hour, clinically presented with arrhythmia, rhabdomyolysis of both skeletal and cardiac muscles, lactic acidosis, renal failure, hypertriglyceridemia, elevated liver enzymes, and finally cardiac arrest [1].

The mechanism behind this syndrome is not fully understood, but it has been thought that propofol induces blockade of the mitochondrial fatty oxidation and accumulation of unutilized free fatty acids with arrhythmic and metabolic fatal consequences. It has also been thought that glucose depletion during RSE may be a precipitating factor to PRIS development [2].

The patients with RSE are amongst the most susceptible group of patients to develop PRIS due to the tendency to use a higher dose of propofol.

## 2. Materials

### 2.1. Case Report

A twenty-eight-year-old Caucasian female, weighing 50 kg, mentally retarded, but with no other dysfunction or any detected genetic abnormalities, had suffered from a refractory form of epilepsy for many years and had been treated in a special neurological unit with multimodal antiepileptic medication regime consisting of carbamazepin, topiramate, and clobazam and stimulatory impulses of the vagal nerve.

The patient had a history of developing tonic-clonic seizures of which there were several episodes during the week before admission. Following admission to the intensive care unit (ICU), the usual antiepileptic medication was supplemented with valproate, phenytoin, and levetiracetam without successfully alleviating seizures. The patient was finally anaesthetized with propofol and remifentanil infusions and intubated to secure airway.

The mean dosage of propofol infusion was 12 mg/kg/h over a period of 78 hours, but the maximal infusion rate was 84 mg/kg/t before successful burst suppression was achieved.

The first symptom suspected to be PRIS manifestation was unexplained metabolic acidosis with increased lactate concentration which initially appeared 48 hours after admission to the ICU. It was decided to decrease the rate of propofol infusion. Blood pressure declined, despite norepinephrine infusion; therefore, propofol infusion was terminated. A possible alternative relying on thiopental infusion was considered, but this treatment option was not achieved.

The ECG changes of ventricular extrasystole and then bradycardia with junctional rhythm occurred 7 to 8 hours after lactate acidosis had developed. Noradrenalin infusion was increased to 1 *μ*g/kg/min to keep MAP above 70 mmHg and adrenaline infusion was also added.

It took about 100 minutes for cardiac arrest (severe bradycardia, right bundle branch block, and then asystole) to occur after propofol infusion was stopped. The patient died after 22 minutes of resuscitation.

### 2.2. Topical View

We searched MEDLINE, Embase, CINAHL, Cochrane Reviews, and ClinicalKey databases for all relevant literatures that describe all events of propofol or propofol-related infusion syndrome reported in cases of refractory SE or SE.

## 3. Results

The collected data has been described and illustrated in Tables 1 and 2.

There are a total of 21 patients: 16 adults and 5 children with RSE described in 16 case reports to develop resuscitation requiring PRIS.

From a total of 16 case reports, there were 3 cases of PRIS who developed cardiac arrest out of a total of 14 patients who developed one or more criteria of PRIS from Iyer et al.'s retrospective study [3], 3 cases from Kumar et al. [4], and 2 cases from Hanna and Ramundo [5] and the rest are single case-based reports [6–18].

As shown in Table 1, most of the patients developed typical presentations of PRIS, reported in different ratios: arrhythmia in all cases, rhabdomyolysis in 9 cases (42%), and lactic acidosis in 13 cases (62%).

Renal failure (low or absent urine output and/or elevated creatinine), hypertriglyceridemia or lipemia, and elevated hepatic enzymes have been described in 8, 7, and 5 patients, respectively.

RSE has been presented as general tonic-clonic status epilepticus (GTCSE), simple partial status epilepticus (SPSE), and nonconvulsive status epilepticus (NCSE) in 9, 4, and 3 patients, respectively.

Interestingly, despite the fact that previously several cases have shown the fatality of propofol given in a dose higher than 4 mg/kg/h over more than 48 h which is the maximal dosage to be supported strongly by the international recommendations, higher doses have still been given in recent reports (Deters et al., Savard et al., and Mayette et al. in 2013 [7, 9, 10] and Chen and Lim in 2014 [17]).

In the case presented by Levin et al. in 2015, the patient has been treated with propofol infusions in a dosage much less than the highest recommended level of 4 mg/kg/h, though the patient still developed clinical symptoms mimicking PRIS, which may make the recommended dosage level of propofol infusion questionable [15].

The mortality rate of PRIS developed in all reported cases is 62%. It is worth mentioning that 6 patients who had been treated with new methods such as CRRT, ECMO, plasma exchange, and blood exchange had all survived, and only 2 had survived after immediate interruption of propofol infusion and received antiarrhythmic treatment with lidocaine (9-month-old infant) and magnesium.

## 4. Discussion

Whether propofol, midazolam, or pentobarbital is the drug of choice in the management of RSE is still a matter of debate. There are very few studies which compare propofol with midazolam and/or pentobarbital.

Stecker et al. retrospectively included 16 patients to compare between propofol and barbiturates and showed that there was no significant difference in mortality. There were more seizures controlled in patients with high-dose barbiturate therapy (82%) than with propofol (63%). The mortality rate was 87.5% with propofol versus 50% with pentobarbital [19].

Prasad et al. compared 14 patients treated with propofol with 6 patients treated with midazolam and concluded that they did not differ in seizure control but mortality was higher in propofol group (57%) than in midazolam group (17%) [20].

However, the attainment of significance in both studies was not possible due to the small sample size of the patients.

In a systematic review, Claassen et al. compared propofol with midazolam or pentobarbital and revealed that adults treated for refractory status epilepticus had a 48% mortality rate regardless of which agent was used [21].

Niermeijer et al. reviewed 74 patients with status epilepticus treated with propofol and included the patients from 2 of the abovementioned studies [21, 22], where 22 patients died, and they concluded that propofol safety in the setting of status epilepticus is questionable and therefore its use should not be recommended [22].

Iyer et al. [3] retrospectively compared 31 patients treated with propofol with 10 patients treated with other medications such as pentobarbital, lorazepam, and midazolam at the same intensive care unit. There were 11 patients in propofol group with one or more PRIS criteria without the need for treatment and 3 patients developed cardiac arrest (2 died and 1 survived), so it was concluded that PRIS develops in a dose dependant manner. No mortality was reported in nonpropofol group, which again brings into question the safety profile of propofol use in RSE.

## 5. Conclusion

The rate of propofol infusion should be carefully titrated and varied recommendations about the maximal infusion rate and period need to be reviewed in light of repeated reports of propofol infusion syndrome associated with high propofol infusion rate during RSE treatment. Propofol infusion syndrome requires early detection and immediate management.

## Figures and Tables

**Table 1:** Clinical presentation and investigations.

	Survived *n* = 7	Died *n* = 14
Sex (female/male)	3/4	2/11
Age (years), mean (range)	21,1 (9 months–55)	36,5 (7–64)
Children (*n*)	2	2
Type of epilepsy, *n* (%)		
NCSE	—	4 (28,6)
SPSE	1 (14,3)	4 (28,6)
CPSE	—	2 (14,3)
GTCSE	4 (57,1)	2 (14,3)
Unknown	2 (28,6)	2 (14,3)
Duration of propofol infusion, hours (±SD)	54,4 (±20,2)	72,2 (±22,2)
Propofol doses (mg/kg/hr), mean (±SD)	8,8 (±4,4)	9,8 (±3,8)
ECG changes		
RBBB	2 (28,6)	3 (21,4)
Brugada	1 (14,3)	1 (7,14)
Wide QRS	2 (28,6)	4 (28,6)
Prolonged QTc	2 (28,6)	3 (21,4)
Sinus bradycardia	2 (28,6)	5 (35,7)
Torsade de Pointes	1 (14,3)	1 (7,14)
Asystole		1 (7,14)
PEA		1 (7,14)
Rhabdomyolysis, *n* (%)	1 (14,3)	8 (57,1)
Lactic acidosis, *n* (%)	5 (71,4)	8 (57,1)
Renal failure, *n* (%)	4 (57,1)	4 (28,6)
Triglyceridemia, *n* (%)	3 (42,9)	4 (28,6)
Elevated liver enzymes, *n* (%)	3 (42,9)	2 (14,3)

NCSE: nonconvulsive status epilepticus; SPSE: simple partial status epilepticus; CPSE: complex partial status epilepticus; GTCSE: general tonic-clonic status epilepticus; RBBB: right bundle branch block; PEA: pulseless electrical activity.

**Table 2:** Treatment and outcome.

	Survived *n* = 7	Died *n* = 14
Treatment, *n* (%)		
Magnesium	—	1 (7,1)
Acidosis correction	2 (28,6)	4 (28,6)
IV fluids	—	5 (35,7)
Glucagon	—	1 (7,1)
Pentobarbital/ketamine	—	1 (7,1)
CRRT	4 (57,1)	1 (7,1)
ECMO	2 (28,6)	—
Therapeutic plasma exchange	1 (14,3)	—
Lidocaine for arrhythmia	1 (14,3)	—
IV calcium	2 (28,6)	—
Partial exchange blood transfusion	1 (14,3)	—
Unknown	—	3 (21,4)
Vasopressor, *n* (%)		
Dopamine	1 (14,3)	6 (42,9)
Dobutamine	—	2 (14,3)
Adrenalin	1 (14,3)	2 (14,3)
Phenylephrine	—	3 (21,4)
Noradrenaline	2 (28,6)	1 (7,1)
Vasopressin	—	1 (7,1)
Milrinone	1 (14,3)	—
Neosynephrine	—	1 (7,1)
Unknown	3 (42,9)	5 (35,7)

CRRT: continuous renal replacement therapy; ECMO: extracorporeal membrane oxygenation.
